# *Trebouxia* *lynnae* sp. nov. (Former *Trebouxia* sp. TR9): Biology and Biogeography of an Epitome Lichen Symbiotic Microalga

**DOI:** 10.3390/biology11081196

**Published:** 2022-08-10

**Authors:** Eva Barreno, Lucia Muggia, Salvador Chiva, Arantzazu Molins, César Bordenave, Francisco García-Breijo, Patricia Moya

**Affiliations:** 1 Instituto Cavanilles de Biodiversidad y Biología Evolutiva (ICBiBE), Departamento de Botánica y Geología, Universitat de València, C/Dr. Moliner, 50, 46100 Burjassot, Spain; 2 Department of Life Sciences, University of Trieste, Via L. Giorgieri 10, 34127 Trieste, Italy or; 3 Instituto de Investigaciones Agroambientales y de Economía del Agua (INAGEA), Departamento de Biología, Universitat de les Illes Balears (UIB), Ctra. Valldemossa Km.7., 07122 Palma de Malllorca, Spain; 4 Laboratorio de Anatomía “Julio Iranzo”, Jardín Botánico de la Universitat de València, C/Quart, 81, 46008 València, Spain; 5 Departamento de Ecosistemas Agroforestales, ETSIAMN, Universitat Politècnica de València, Camino de Vera s/n, 46022 València, Spain

**Keywords:** culture, diversity, genetics, isolation, morphology, phylogeny, *Ramalina*

## Abstract

**Simple Summary:**

In this work, we present the formal description of a new species of lichen photobiont (i.e., *Trebouxia lynnae*) isolated from the lichen *Ramalina farinacea*. The findings reported here provide an exhaustive characterization of the cellular ultrastructure, physiological traits and genetic and genomic diversity of the new species. Our results contribute to the knowledge of lichen-forming symbiotic green microalgae with their diversity and distribution.

**Abstract:**

Two microalgal species, *Trebouxia jamesii* and *Trebouxia* sp. TR9, were detected as the main photobionts coexisting in the thalli of the lichen *Ramalina* *farinacea*. *Trebouxia* sp. TR9 emerged as a new taxon in lichen symbioses and was successfully isolated and propagated in *in vitro* culture and thoroughly investigated. Several years of research have confirmed the taxon *Trebouxia* sp. TR9 to be a model/reference organism for studying mycobiont–photobiont association patterns in lichen symbioses. *Trebouxia* sp. TR9 is the first symbiotic, lichen-forming microalga for which an exhaustive characterization of cellular ultrastructure, physiological traits, genetic and genomic diversity is available. The cellular ultrastructure was studied by light, electron and confocal microscopy; physiological traits were studied as responses to different abiotic stresses. The genetic diversity was previously analyzed at both the nuclear and organelle levels by using chloroplast, mitochondrial, and nuclear genome data, and a multiplicity of phylogenetic analyses were carried out to study its intraspecific diversity at a biogeographical level and its specificity association patterns with the mycobiont. Here, *Trebouxia* sp. TR9 is formally described by applying an integrative taxonomic approach and is presented to science as *Trebouxia lynnae*, in honor of Lynn Margulis, who was the primary modern proponent for the significance of symbiosis in evolution. The complete set of analyses that were carried out for its characterization is provided.

## 1. Introduction

Lichens are iconic examples of symbiotic interactions originated by the living together of heterotrophic ascomycetous or basidiomycetous fungi (i.e., the mycobionts) and populations of photosynthetic green microalgae (phycobionts) or cyanobacteria (cyanobionts) (i.e., the photobionts). Aside from these two major lichen symbionts that shape their unique symbiosis into a thallus, an indeterminate number of other microscopic organisms co-occur, intermingled in these associations [1,2,3].

Among the phycobionts, the genus *Trebouxia* Puymaly (*Trebouxiaceae*) is the most common and associates with a broad spectrum of lichen-forming ascomycetes worldwide [4,5,6,7,8]. Muggia et al. [6] assembled the most comprehensive taxon sampling for *Trebouxia* and provided a genus-wide, multi-locus phylogenetic hypothesis to use as a reference in subsequent studies. In their study the authors confirmed the recognition of four main *Trebouxia* clades—clade A [*Trebouxia arboricola* Puymaly/*Trebouxia gigantea* (Hildreth & Ahmadjian) Gärtner group], clade C [*Trebouxia corticola* (P.A.Archibald) Gärtner/*Trebouxia galapagensis* (Hildreth & Ahmadjian) Gärtner group], clade I (*Trebouxia impressa* Ahmadjian/*Trebouxia gelatinosa* Ahmadjian ex P.A.Archibald group), and clade S (*Trebouxia simplex* Tschermak-Woess/*Trebouxia suecica* Beck group). Shortly after, Xu et al. [9] suggested the presence of a further, well-supported, monophyletic new *Trebouxia* lineage, named clade D.

The number of species-level lineages recognized in *Trebouxia* increases as soon as new ecological niches or different lichen symbioses are investigated (e.g., [10,11,12]). However, only 30 *Trebouxia* species-level lineages have so far been formally described based on their cell morphology/ultrastructure and genetic diversity [6,13,14,15,16]. Indeed, a reliable species identification and characterization is achieved only when *Trebouxia* cells grow *in vitro* outside the symbiotic state of the lichen thallus. Key diagnostic features of chloroplast morphology and pyrenoid ultrastructure develop at their best when the algae are axenically grown in cultures. Unfortunately, thus far, still too few species have been successfully isolated [14]. Furthermore, isolation and culture approaches for *Trebouxia* were only standardized a few years ago [17,18,19], while only recently, Bordenave et al. [15] delivered a detailed morphoanatomical characterization of pyrenoid and chloroplast structures. The authors recognized six types of pyrenoid ultrastructure and five main types of chloroplasts among the 20 *Trebouxia* species-level lineages to be used as a reference for species identification [15]. Garrido-Benavent et al. [16] subsequently added a new maresiae-type of pyrenoid.

In the past decades, several studies have proven the coexistence of multiple *Trebouxia* species-level lineages within a single lichen thallus, shedding light on diverse patterns of photobiont–mycobiont associations [20,21,22,23,24,25,26]. The pioneer studies in this field were those of del Campo et al. [27,28], which revealed the coexistence of multiple *Trebouxia* taxa in the individual thalli of the lichen *Ramalina farinacea*. While most of these microalgae were identified as *Trebouxia jamesii* (the most abundant phycobiont in that thalli), the microalga numbered *Trebouxia* sp. TR9 was genetically and ultrastructurally different. The discovery of the presence of two photobionts in a single thallus made the lichen *R. farinacea* a model/reference system to study the photobiont coexistence in lichens (e.g., [10,11,29,30]). The application of the DNA metabarcoding approach corroborated the coexistence of the two photobionts (*T. jamesii* and *Trebouxia* sp. TR9) in individual thalli of *R. farinacea*, but also highlighted a much higher, unexpected microalgal diversity [31]. Taking *R. farinacea* as a reference system, Molins et al. [32] pursued the reappraisal of the microalgal diversity in thalli from different ecologies by performing an ad hoc sampling design and an in-depth Illumina paired end metabarcoding approach. Their results show that in many cases, there is no balanced co-presence of *T. jamesii* and *Trebouxia* sp. TR9, as previously determined in *R. farinacea* [32].

Since then, *Trebouxia* sp. TR9 and *Trebouxia jamesii* were recurrently found in the thalli of both *R. farinacea* and other lichen species using culture isolations and Sanger sequencing (e.g., [22,23,27,28,29,31,32]). These species have been successfully maintained as viable *in vitro* culture for over 10 years. Several studies performed with *Trebouxia* sp. TR9 have generated an exhaustive knowledge on this alga, aiming at its characterization. This microalga presents a pyrenoid impressa-type and a shallowly lobed-type of chloroplast [15]. It is photosynthetically better performing at higher temperature and irradiance [22], and shows novel inducible responses against abiotic stresses. In particular, *Trebouxia* sp. TR9 responds well against oxidative stress [33], which is a crucial challenge for lichens exposed to cyclic desiccation and rehydration events, nitric oxide (NO) [34,35,36,37,38], osmotic and saline stresses [39,40], and photooxidants or heavy metal accumulation [41,42,43]. The better physiological performance of *Trebouxia* sp. TR9 under oxidative conditions than that of the coexisting *T. jamesii* may reflect its greater capacity to undertake key metabolic adjustments including increased non-photochemical quenching, higher antioxidant protection, and the induction of repair mechanisms [22]. Additionally, *Trebouxia* sp. TR9 generate peaks of NO-end-products in suspension and show high rates of photobleaching and reactive oxygen species (ROS) production under NO inhibition. NO is indeed a key molecule, conferring stress tolerance in lichens during early stages of thallus rehydration [34,41]. Hinojosa-Vidal et al. [39] studied the effects of prolonged exposure to high salt concentrations on *Trebouxia* sp. TR9 and demonstrated that this microalga displays a rather different molecular response compared to land plants and free-living halophilic microalgae. *Trebouxia* sp. TR9 does not significantly increase the abscisic acid (ABA) levels and ABA-related gene expression until the external NaCl concentration is raised to 3 M NaCl. Furthermore, the responses of *Trebouxia* sp. TR9, *Asterochloris erici*, and *Chlorella vulgaris* to osmotic and saline stresses were compared and *Trebouxia* sp. TR9 had an extraordinarily higher tolerance to osmotic and saline stress than the other two species [40]. This suggests that *Trebouxia* sp. TR9 may have developed alternative molecular pathways to cope with highly saline environments.

Another property of *Trebouxia* sp. TR9 is the capacity to immobilize most metals extracellularly such as when exposed to Pb, while in *T. jamesii*, the amount of intracellular Pb accumulation is three times higher than in *Trebouxia* sp. TR9 [44,45]. Both phycobionts adopt two different strategies against Pb stress (*Trebouxia* sp. TR9 forms extracellular aggregates, while *T. jamesii* has a lower wall Pb retention capability), in which the integration of distinct anatomical and physiological features affords similar levels of Pb tolerance [45,46]. Related to this result, cell walls and extracellular polymers from *T. jamesii* and *Trebouxia* sp. TR9 were studied. The proportion of cell walls on the overall cell biomass was 2.6 times higher in *Trebouxia* sp. TR9 than in *T. jamesii* [45]. At the ultrastructural level, four clearly differentiable layers in the *T. jamesii* cell wall were observed, whereas *Trebouxia* sp. TR9 showed a more diffuse structure in which only three layers could be distinguished. In general, cell wall biomass, monosaccharide composition, and extracellular polymers of *Trebouxia* sp. TR9 and *T. jamesii* showed clear differences, suggesting close associations between the differential ultrastructure and Pb-retention capabilities [44,45,46].

The genetic characterization of *Trebouxia* sp. TR9 was completed by sequencing its nuclear and organelle genomes using the Illumina, 454, and Solexa sequencing technologies [47,48,49,50]. The nuclear genome of *Trebouxia* sp. TR9 (59.7 Mb) covers 100% of the estimated genome size and has a completeness of 96.7%. The number of detected gene models was 15,905, and the functional annotation had been improved with a total of 7068 different GO terms, 1826 enzyme class terms, and 7581 different gene annotations [47,48]. The mitochondrial genome sequence of *Trebouxia* sp. TR9 was the first complete mtDNA genome sequence available for a lichen-symbiont microalga [49]. It comprises 70,070 bp and has a total of 61 genes; nine type I introns were detected in several genes. The chloroplast genome of *Trebouxia* sp. TR9, instead, comprises 303,323 bp, resulting in one of the largest known genomes among Chlorophyta [50]. A total of 108 genes and 12 type I introns have been detected. The most remarkable characteristics are the presence of long intergenic spacers, the typical quadripartite structure of land plant chloroplasts with short inverted repeated sequences (IRs), a single gene of rbcL, and the loss of the rps4 gene, which was transferred from the chloroplast to the nucleus. Currently, whole genome sequencing offers new ways to study lichen populations and their interaction with their environments. Furthermore, phylogenomics analyses can help in resolving closely related or recently diverged lineages.

Lichens are poikilohydric organisms and limit the photosynthetic CO_2_ assimilation to relatively short periods of time when their thalli are sufficiently hydrated, and the photobionts are metabolically active [51,52,53]. Isotopic discrimination is a widely used technique in cyanobacteria and microalgae to determine the presence or absence of a carbon-concentrating mechanism (CCM) [54,55,56,57]. A CCM can provide a rapid response mechanism in environments where light and CO_2_ availability are limited [58,59]. Moreover, the activity of a CCM may also be related to the nitrogen economy of the organism. A lichen with a CCM may need to invest less in both the carboxylating enzyme Rubisco, and enzymes involved in the recovery of assimilates during photorespiration [60,61,62]. Traditionally, physiological studies of isotopic discrimination in algae or bryophytes have indicated that pyrenoids are related to CCM [54,62,63,64]. The identification of proteins involved in carbon uptake suggests that *Trebouxia* sp. TR9 may possess carbon concentration mechanisms similar to C3 and C4/CAM [47].

*Trebouxia* sp. TR9 is evidently one of the best analyzed lichen-forming microalgae but thus far, it has not been formally described. Here, we accomplish its formal description following an integrative taxonomic approach by merging all the information gained in several previous works and the present one. We propose for this taxon, the name *Trebouxia lynnae* Barreno sp. nov., in honor of the outstanding biologist Lynn Margulis, who was the primary modern proponent for the significance of symbiosis in evolution, in her words: “Life is a symbiotic and cooperative union that allows those who partner together to succeed”. In doing this, we also provide an overview of the methodologies that were applied herewith and in the past years.

## 2. Materials and Methods

### 2.1. Isolation and Cultivation of Phycobionts

Lichen thalli of *Ramalina farinacea* (L.) Ach were collected in 2006 from El Toro, Castellón, Spain (39°57′32.34″ N, 0°46′35.51″ W) at 1150 m. Of these, one specimen was dried for one day and stored at −20 °C until processing. Thallus laciniae were examined under a stereomicroscope to remove the surface contamination (e.g., sand, epiphytic algae, fragments of mosses or other lichen species, or infection by lichenicolous fungi). Clean laciniae were homogenized with a mortar and pestle in an isotonic buffer (0.3 M sorbitol, 50 mM HEPES, pH 7.5) and filtered through muslin. Isolation was carried out by a gradient centrifugation method using Percoll^®^, as described in Gasulla et al. [17]. The algal suspension was diluted with sterile water and 10 μL was spread using the streak method on sterile 1.5% agar Bold’s Basal Media Petri dishes (BBM) [65,66]. Once algal colonies grew to a sufficient biomass (about 1 mm wide colony), they were individually sub-cultivated on liquid BBM (named as unialgal culture). A subsample of this liquid culture was taken for DNA extraction and genetic identification. A volume of 50 μL of this unialgal culture was plated over solid BBM for 21 days [18] and the grown colonies were used for morphological analyses. Moreover, 50 μL of this unialgal culture was transferred on liquid 3N BBMGC under standard conditions—21 days at 20 °C—used for isotopic discrimination. This unialgal culture was stored as both a living strain and cryostocks as ASUV44 at the Symbiotic Algal Collection of the Universitat de València (ASUV) and at the BEA 2029B (Banco Español de Algas) culture collections. Conditions for the viable cultures were kept in a growth chamber under the following conditions: 20 °C, 12:12 h light:dark cycle (25 μmol photons m^−2^ s^−1^).

### 2.2. DNA Extraction, Amplification, and Sequencing

Total genomic DNA of the unialgal liquid culture was isolated and purified using the DNeasy TM Plant Mini Kit (Qiagen, Hilden, Germany) following the manufacturer’s instructions. Three algal loci were amplified for a multi-locus *Trebouxia* phylogeny using the same algal-specific primers established by Muggia et al. [6]. These molecular markers are: the rDNA internal transcribed spacer (ITS), the ribulose-1,5-bisphosphate carboxylase/oxygenase large subunit (rbcL), and the cytochrome c oxidase (cox2). The algal nuclear ITS region (ITS1, 5.8S, ITS2) was amplified with the primers ITS1T and ITS4T [67]; the plastidial rbcL was amplified using primers rbcL151f and rbcL986R [68]; the mitochondrial cox2 locus was amplified with the primers COXIIf2 and COXIIr [69]. PCR reactions and Sanger sequencing were performed as described in Molins et al. [19]. The PCR products were visualized on 1% agarose gels and purified using the Gel Band Purification Kit (GE Healthcare Life Science, Logan, UT, USA). Complementary strands of the cleaned PCR products were sequenced by Sanger sequencing (http://www.stabvidacom/, accessed on 1 July 2022) with the same primers used for amplification.

### 2.3. Phycobiont Phylogenetic Analysis

A total of 200 *Trebouxia* sequences were used for the alignments: 91 ITS, 46 cox2, and 63 rbcL (Supplementary Table S1). A multiple sequence alignment (MSA) for the ITS was constructed including the *Trebouxia* clade A dataset updated with recent published species-level lineages or known species (n = 89) and two selected sequences from clade S [6] included as the outgroup. The MSA was built in MAFFT v 7.0 [70,71] using the default parameters. The substitution model GTR + I + G was the most accurate for the ITS region according to the Akaike information criterion (AIC) using JModelTest v 2.1.4 [72]. Multiple sequence alignments of the protein-coding rbcL and cox2 sequences were relatively conserved, and both regions were aligned following the same methodology. The substitution models GTR + I + G and HKY + G were the most accurate for rbcL and cox2, respectively.

Phylogenetic relationships were inferred from the concatenated multi-locus sequence dataset (ITS, rbcL and cox2) by Bayesian inference (BI) and maximum likelihood (ML) approaches. ML analysis was implemented in RAxML v 8.1.11 [73] using the GTRGAMMA substitution model. Bootstrap support (BS) was calculated based on 1000 pseudoreplicates [74]. BI was carried out in MrBayes v 3.2 [75]. Settings included two parallel runs with six chains over 20 million generations, starting with a random tree and sampling after every 200th step. We discarded the first 25% of data as burn-in, and the corresponding posterior probabilities (PPs) were calculated from the remaining trees. Estimated sampled sized (ESS) values above 200 and potential scale reduction factor values approaching 1000 were considered as indicators of chain convergence. All analyses were performed with the CIPRES Science Gateway v 3.3 [76]. Phylogenetic trees were visualized in FigTree v 1.4.1 [77].

### 2.4. Microscopic Analyses of Phycobionts Grown in Culture

A volume (50 μL) of the unialgal culture were plated over solid BBM for 21 days [18] and the grown colonies were used for morphological analyses. We referred to the original classifications of Friedl et al. [13] and to the most recently compiled revision conducted by Bordenave et al. [15] for the characterization of both chloroplast and pyrenoid types.

Light microscopy (LM) and differential interference contrast (DIC) microscopy were used to study the morphological traits of algal cells using a NIKON ECLIPSE E-800 microscope. Algal cells were picked from the colony and mounted in water, gently pressing on the cover slide. The samples were photographed with a NIKON DS-RI1 camera (ICBIBE Microscopy Service, Paterna, Spain).

Transmission electron microscopy (TEM) was applied to study the ultrastructural traits of pyrenoid and chloroplast. The cells were fixed and dehydrated as described in Molins et al. [26]. The samples were embedded in Spurr’s resin according to the manufacturer’s instructions. Ultra-thin sections (80 nm) were cut, mounted, and stained with 10% uranyl acetate and 0.1% lead citrate using the ‘Synaptek Grid-Stick Kit’, as described in Moya et al. [78]. The original lichen thalli were fixed and treated as described for the axenic cultures. The ultrathin sections were observed with a JEOL JEM-1010 (80 kV) electron microscope, equipped with a MegaView III digital camera and ‘AnalySIS’ image acquisition software (Olympus, Tokyo, Japan) (SCSIE, Universitat de València).

Confocal laser scanning microscopy (CLSM) algal colonies were embedded in temperate 1% low melting point agarose and the first section was obtained by performing two parallel vertical cuts with a sharp blade. The section was embedded again in agarose and sectioned in half with a cut parallel to the previous ones. One of the half sections was placed with the sectioned side face down over a 35 mm imaging dish suitable for inverted microscopy. An Olympus FLUOVIEW FV1000 laser scanning confocal microscope was used with a 488 nm excitation laser. Fluorescence emitted from 650 to 750 nm was collected to observe the chlorophyll autofluorescence, thus recovering the chloroplast layers. A series of images were captured with a separation of 0.4 µm. The image stack was preprocessed to remove noise and then analyzed using the z-projection tool and volume viewer with Fiji distribution in ImageJ [79].

For cryo-scanning electron microscopy (cryo-SEM), algal colonies were fixed on a holder, frozen in nitrogen sludge, and transferred in a carrier into a cryo-SEM system (PP3010T, Quorum Technologies). The samples were mechanically fractured and then sublimated for 15 min at −90 °C. The samples were sputtered with a thin layer of platinum for 10 s and transferred afterward into the SEM (FESEM ZEISS Ultra-55). Images were recorded at an accelerating voltage of 1.5 kV.

### 2.5. Isotopic Discrimination

*Trebouxia* sp. TR9 and *T. jamesii* cultures grew under standard conditions (i.e., 21 days in liquid 3NBBMGC at 20 °C) [18]. These cultures were used to determine the carbon (δ^13^C) and nitrogen (δ^15^N) isotopic compositions and content. Samples were dried for 48 h in an oven at 60 °C and ground into fine powder. Subsamples of 2 mg were combusted in an elemental analyzer (Thermo Flash EA 1112 Series, Bremen, Germany), and CO_2_ and N_2_ were directly injected into a continuous-flow isotope ratio mass spectrometer (Thermo- Finnigan Delta XP, Bremen, Germany) for isotope analysis. Peach leaf standards (NIST 1547) were run every six samples. The standard deviation of the analysis was below 0.1‰. Results are presented as δ vs. PDB (referred to a Pee Dee Belemnite standard) for δ^13^C, and δ vs. SMOW (referred to standard median ocean water) for δ^15^N. Nitrogen content was calculated from the area obtained for isotope analysis on mass 28 and carbon on mass 44.

## 3. Results

**Taxonomy:**

- Family *Trebouxiaceae* Friedl
- Genus *Trebouxia* Puymaly
- ***Trebouxialynnae* Barreno sp. nov.**

Ethymology: The species is named in honor of the outstanding evolutionist Lynn Margulis, who encouraged Eva Barreno to study symbiotic microalgae and bacteria using new techniques.

Description: Mostly unicellular, but also grouped in tetrads and octads. Vegetative mature cells spherical 7–12 (16) μm in diameter. Cell wall composed of three layers. Chloroplast shallowly lobed-type with a central mass from which elongated lobes arise and meander around the chloroplast surface. Chloroplasts with one or more impressa-type pyrenoids, characterized by radial, straight, and unbranched tubules penetrating the pyrenoid matrix, appearing either as long or short depending on the orientation of the section; starch granules are often embedded in the chloroplast stroma. The pyrenoid matrix is always thicker than the tubules; pyrenoglobules are always present in moderate numbers inside the matrix, arranged in a row next to the invaginations of the tubules. Asexual reproduction with the formation of 4–32 autospores within utosporangia (10–18 μm) of an irregular shape; autospores are tightly appressed to each other. Sexual reproduction with zoospores, biflagellate with oval (3.5 × 5 μm) cells (Table 1).

Diagnosis: Differing from other *Trebouxia* species in clade A by the pyrenoid and chloroplast type. Usually, *Trebouxia* species in clade A present only one single pyrenoid per chloroplast, except for *T. jamesii* and *T. lynnae* sp. nov., which contain one or more impressa-type pyrenoids. *T. lynnae* sp. nov. clearly differs from the other accepted *Trebouxia* species (Table 1) including *T. jamesii* in the ITS and the rbcL and cox2 molecular markers. *T. lynnae* sp. nov. appears as a sister species of *T. jamesii*, sharing the pyrenoid and chloroplast type. It has been widely referenced in the literature as *Trebouxia* sp. TR9.

Type locality: Phycobiont in *Ramalina farinacea*, collected on *Quercus rotundifolia* Lam. at Sierra El Toro (Castellón, Spain; 39°57′32.34″ N, 0°46′35.51″ W at 1150 m, leg. E. Barreno and F. Gasulla). Bioclimate low supramediterranean (ITC0203) low subhumid (IO = 4.7) [85].

Holotype (designated here): Cryopreserved cells of strain *T.* *lynnae* sp. nov. deposited at the Symbiotic Algal collection at the Universitat de València (ASUV), as item TYPE–ASUV 44.

Reference strains: ASUV 44 and BEA 2029B.

**Figure 1:**
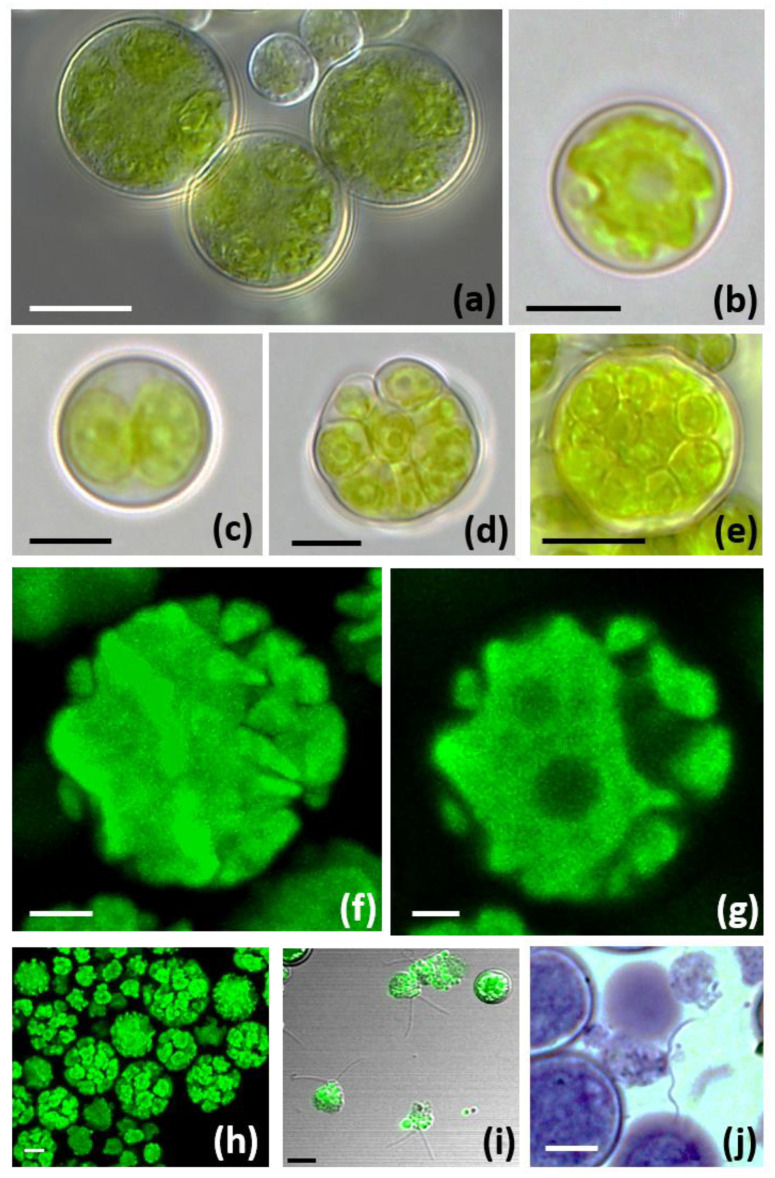
The morphology of *Trebouxia lynnae* sp. nov.: (**a**) Image composition of *T. lynnae* cells using differential interference contrast microscopy; (**b**) light micrographs (LM) of mature vegetative cell, and (**c**–**e**) autosporangia containing different number of autospores; (**f**,**g**) confocal microscopy reconstructions of the mature chloroplast with shallow, elongated lobes and (**h**) several autosporangia. (**i**,**j**) Zoospores. Scale bars: (**a**–**e**,**j**) =10 μm, (**f**–**h**) =5 μm, (**i**) =20 μm. Reference strain: ASUV 44.

**Figure 2:**
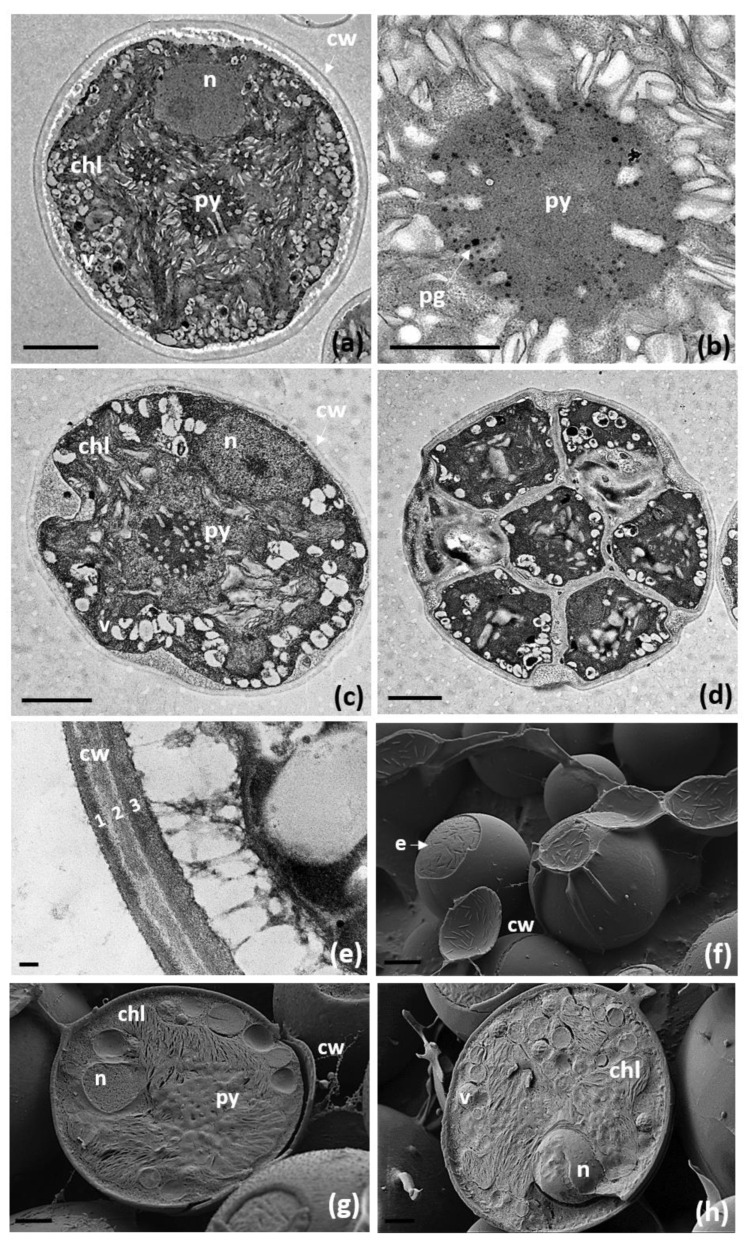
The TEM micrographs of the ultrastructure of *Trebouxia lynnae* sp. nov. in culture: (**a**,**c**) *T. lynnae* cells; (**b**) detail of pyrenoid; (**d**) aplanospore (**e**) detail of the cell wall containing three layers (1,2,3); (**f**–**h**) cryo-SEM images of the entire (**f**) and broken cells (**g**,**h**), arrow points at the eisosome attached to the outer layer of the plasma membrane (**f**). Abbreviations: chl (chloroplast), cw (cell wall), e (eisosomes), n (nucleous), Py (pyrenoid), and v (vesicles). Scale bars: (**a**) =2 μm, (**b**) =1 μm, (**c**,**d**) =2 μm, (**e**) =100 nm, (**f**) =2 μm, (**g**,**h**) =1 μm. Reference strain: ASUV 44.

**Figure 3:**
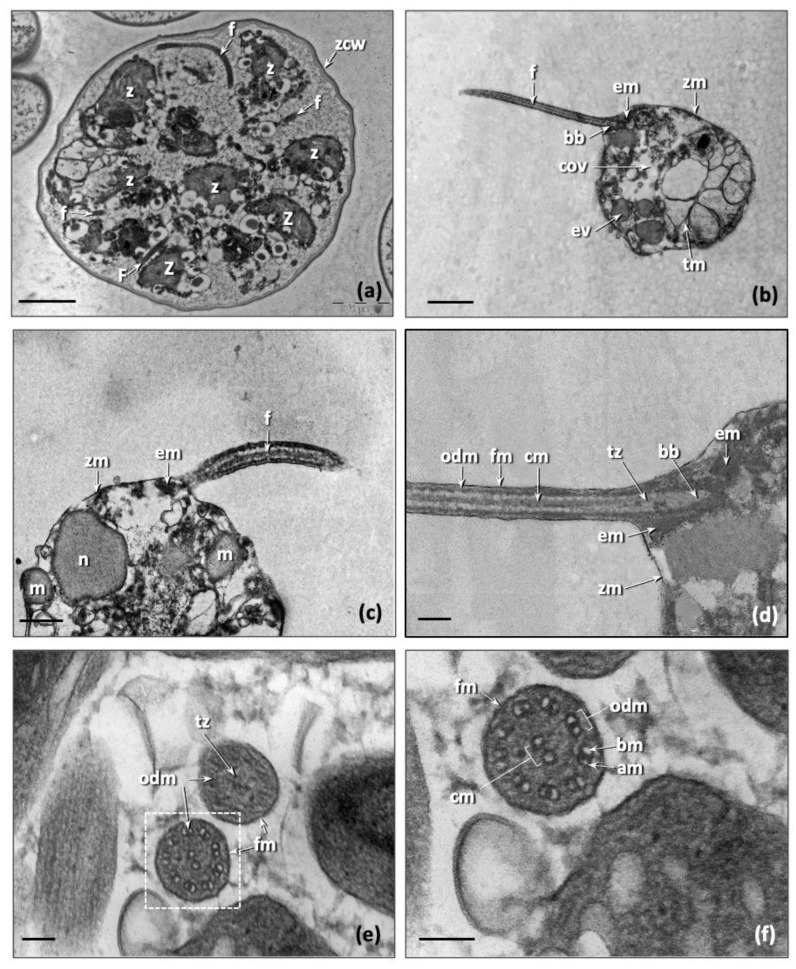
The zoospores of *Trebouxia lynnae* sp. nov.: (**a**) zoosporangium with several zoospores; (**b**,**c**) longitudinal section of a zoospore; (**d**) longitudinal view of the flagella insertion in the zoospore; (**e**) cross section of two flagella in a young zoospore, one in the filament zone and another in the transition zone; (**f**) detail of the filament zone flagella. Abbreviations: am (microtubule a), bb (basal body), bm (microtubule b), cm (central microtubules), cov (contractile vesicle), em (electrodense material), ev (electrondense vesicle), f (flagellum), fm (flagellum membrane), m (mitochondria), n (nucleus), odm (outer microtubules doublet), tm (thylakoidal membrane), tr (transition region), tz (transition zone), zcw (zoosporangium cell wall), z (zoospore), zm (zoospore membrane). White arrows point to the peculiar ultrastructures identified by the abbreviations. Scale bars: (**a**) =2 μm, (**b**) =1 μm, (**c**) =400 nm, (**d**) =200 nm, (**e**,**f**) =100 nm. Reference strain: ASUV 44.

Iconotype (designated here to support the holotype): Figure 1, Figure 2 and Figure 3 in this study.

Holotype DNA sequences: KU716051 (nrITS), QHO63910 (rbcL), and QES94804 (cox2). NC_045839 LSU rDNA. The complete mitochondrial and chloroplast genome sequences were deposited under the GenBank accession number MH917293 and MK643158, respectively [49,50].

Ecology and distribution: Bipolar, more frequent in the Northern Hemisphere (Supplementary Table S2).

### 3.1. Phycobiont Phylogenetic Analysis

**Figure 4:**
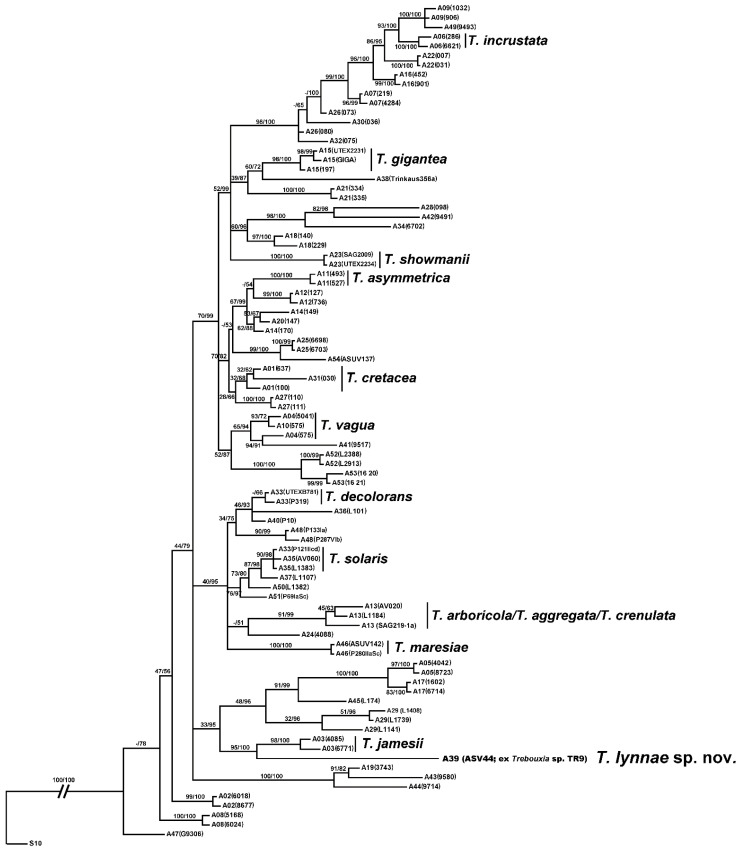
The phylogenetic tree of *Trebouxia* clade A. Rooted tree based on the concatenated ITS, cox2 and rbcL dataset representing 55 *Trebouxia* species-level lineages including sequences (retrieved from the GenBank) of 12 formally described *Trebouxia* species stored at the algal collections SAG and UTEX. *Trebouxia* *lynnae* sp. nov. is highlighted. Values at nodes indicate statistical ML bootstrap supports and Bayesian posterior probabilities (BS/PP). Scale bar shows the estimated number of substitutions per site.

The new species *Trebouxia* *lynnae* belongs to *Trebouxia* clade A (as shown here and in previous studies [5,6,9,10,31,86]) and was found to be a sister species of *T.* *jamesii* (Figure 4). BI and ML analyses were topologically congruent and high posterior probabilities (PP) and bootstrap supports (BS) were obtained for all species-level lineages. The phylogeny inferred is concordant with the reference species-level phylogenies presented by Muggia et al. [6].

### 3.2. Geographical Occurrence

BLAST searches against the GenBank database for the complete or partial ITS were performed. Sequences obtained with 100–99% of identity were downloaded, and the identity was corroborated by phylogeny. A total of 44 sequences were clustered with *Trebouxia lynnae* sp. nov. Two of these were deposited as *T.* *jamesii* and a third as *T.* *decolorans*. Nine were included as uncultured *Trebouxia* and the remaining as *Trebouxia* sp. or *Trebouxia* sp. TR9 (Supplementary Table S2). According to the information reported in GenBank accessions for the field “origin”, *Trebouxia lynnae* sp. nov. was detected as major phycobiont in the following lichen species: *Lecanographa amylacea*, *Protoparmelia montagnei*, *Ramalina decipiens* group, *R. farinacea*, *R. fastigata*, *R. menziesii*, and *Ramalina* sp. These GenBank accessions are reported from Sweden, Poland, North America, New Zealand, Madeira, Cape Verde, and Spain (including the Iberian Peninsula, Canary and Balearic Islands). *T.* *lynnae* sp. nov. was also detected by metabarcoding in the phycobiomes of *Buellia zoharyi*, *Circinaria gyrosa*, and *Circinaria oromediterranea* from the Iberian Peninsula and Balearic Islands.

### 3.3. Morphological and Ultrastructural Characterization of Trebouxia lynnae sp. nov. in Culture

Trebouxia lynnae sp. nov. is characterized by regular coccoid cells of about 7–12 (16) μm in diameter (Figure 1a,b), which at maturity form autospores (Figure 1c–e,h). The autosporangia (10–18 μm) of irregular shape usually contain 4–32 autospores, tightly appressed to each other. In LM and CLSM, the chloroplast occupies almost the whole volume of the cytoplasm and forms shallow, elongated lobes (Figure 1f,g), recalling the ‘shallowly lobed’ type of chloroplast [15]. Zoospores are biflagellate with oval (3.5 × 5 μm) cells (Figure 1i,j).

The TEM analyses of the chloroplast and pyrenoid structures confirmed the presence of an impressa-type pyrenoid (Figure 2a–c). This pyrenoid is characterized by radial straight unbranched tubules penetrating the pyrenoid matrix, appearing as either long or short depending on the orientation of the section. The pyrenoid matrix is always thicker than the tubules. Pyrenoglobules are always present in moderate numbers arranged in a row next to the invaginations of the tubules. In a few cells, more than one pyrenoid was detected (Figure 2a). The nucleus was confined at one side of the cell, likely occupying the biggest invagination in which the chloroplast folds (Figure 2a,c,g,h). Numerous vesicles and starch grains surrounding the pyrenoid were observed (Figure 2a,c,h). The cell wall was composed of three layers (Figure 2e). Cryo-SEM revealed prominent subcellular structures at the cell wall of the microalgae, considered to be eisosomes [87] (Figure 2f).

Zoospores (z) developed inside the zoosporangia, where the flagella were easily observed (Figure 3a). A single contractile vacuole was located in the ventral region of the cell (Figure 3b), and the nucleus laterally (Figure 3c). The thylakoid membranes were disaggregated, and some mitochondria were evident in the cell. Both the flagella of the zoospore were naked and of equal length, approximately 3.3 ± 0.1 μm and had a diameter of 218.2 ± 10.0 nm. The flagellar shaft contained the usual 9 + 2-axoneme that lacks the dynein arms on microtubule of the outer microtubule doublet (Figure 3f). This characteristic feature was described by Melkonian [88] for the motile cells of green algae. The two central microtubules were not surrounded by an inner sheath (Figure 3e,f). The transition zone was very small (Figure 3d). At the axillary junction of the flagellum, close to the basal body, electron-dense material attached to the plasma membrane were observed (Figure 3c,d). The basal body of the zoospores was 350–400 ± 9.9 nm long.

### 3.4. Isotopic Discrimination

The isotopic composition of *Trebouxia lynnae* sp. nov. was −16.96 for δ^13^C and 6.93 for δ^15^N compared to −17.60 and 6.43, respectively, for *T. jamesii* (Table 2). The C/N ratio for *Trebouxia lynnae* sp. nov. and *T. jamesii* was 7.41 and 8.51, respectively, despite the content of both C and N being higher in *Trebouxia lynnae* sp. nov. (Table 2).

## 4. Discussion

We here formally describe a new microalga species of lichen phycobionts (i.e., *Trebouxia lynnae*) and provide a complete set of analyses, which can be taken as a reference for the species description of lichen phycobionts in lichenology. This achievement is a result of several years of research that have seen the success of different techniques to isolate, propagate, and analyze axenically lichen-forming microalgae in culture.

Pure cultures of symbiotic algae are essential for species delimitation following an integrative taxonomic approach in which different analytical methods, combining morphology and genetic diversity, are considered [16,26,31,83,89,90]. Indeed, particularly for the genus *Trebouxia*, the lack of axenically cultured species has been, thus far, one of the major reasons for the unbalanced proportion between the genetically identified species-level lineages and the formally described species [5,6,15,16]. The feasibility of isolating lichen phycobionts was also hindered by the lack of standardized protocols, which instead have nowadays been established and published [15,18,19]. These have so far mainly been applied to study species diversity for the two most frequent lichen-forming genera *Asterochloris* and *Trebouxia* [15,16,89,91], but can be applied alike for the isolation of other less common phycobionts. The isolation procedure used for *Trebouxia lynnae* was based on the Percoll^®^ gradient [17] because this was chosen as the simplest and most cost-effective method for the isolation and cultivation of algal strains in our laboratories. This method can be successfully applied for the isolation of many other coccoid, symbiotic green microalgae (such as *Asterochloris*, *Myrmecia*, *Symbiochloris* or *Vulcanochloris*), photobionts of lichens with diverse growth forms. However, the success rate of microalgae isolation is in general rather low, and it mostly depends on the species-specific requirements of the taxon to be isolated axenically *in vitro* [14]. Several attempts are usually required, and multiple inocula are set before the targeted strain/taxon is successfully isolated. The isolated strains must be genetically identified before proceeding with further analyses; indeed, many morphological traits can be shared by more than two species (as shown in [15]). In this context, *Trebouxia lynnae* represents an exception: it did not require any ad hoc setup to be isolated and it represents a taxon that also grows easily on very poor media (such as BBM, on which it is usually isolated).

Phylogenomics data generated from pure algal cultures are preferred today, rather than metagenomics DNA extracted from lichen thalli to reconstruct robust phylogenies (thereby avoiding amplification of co-existing algae). However, previous multi-locus phylogenetic reconstructions have been essential to uncover the relationships among species-level lineages and their geographic and symbiotic origins in *Trebouxia*. Indeed, *T. lynnae* is known to associate with lichen fungi belonging to phylogenetically distant families such as *Parmeliaceae, Caliciaceae, Lecanographaceae, Megasporaceae*, and *Ramalinaceae* and from different continents and ecologies. Furthermore, *T. lynnae* is the primary phycobiont of lichens that develop diverse growth forms such as the crustose genera *Lecanographa* and *Protoparmelia*, and the fruticose *Ramalina*, and was detected as a minor symbiotic partner in the terricolous crustose *Buellia* and vagrant *Circinaria* from Spain [26,92]. In general, *T. lynnae* is a cosmopolitan species of phycobiont, as it was reported in lichens from Sweden, Poland, North America, New Zealand, Madeira, Cape Verde, and Spain (including the Iberian Peninsula, Canary and Balearic Islands; see Supplementary Table S2 and references therein). The wide versatility of associations and the geographic breadth of *T. lynnae* is consistent with other taxa placed in clade A of *Trebouxia*. In fact, this clade is the one gathering the greatest *Trebouxia* diversity in terms of mycobiont-species associations, geographic origins [5,6,7,8,93,94], and morphological and ultrastructural diversity [15]. Clade A indeed was recently inferred to be the clade that originally, exclusively, or partially, occupied forested habitats, and was subsequently extended to occupy regimes characterized by cooler and drier habitats [7,8], and is now the clade showing the widest distribution among lichen symbioses.

More advanced techniques have been also applied to better investigate the cellular structure: recently, it was shown that fast-freeze electron microscopy techniques (such as cryo-SEM) capture organisms at high resolution in their living state, offering novel views of the cellular ultrastructure, organization, and differentiation [95,96,97]. Although these techniques bypass possible ultrastructure modifications due to preparation procedures, they have not been extensively used with lichens and their axenically isolated phycobionts [96,98]. In the present study, we applied for the first time the cryo-SEM method to the analysis of isolated lichen-forming microalgae. This method allowed us to observe the presence of eisosomes in *T. lynnae* for the first time. Eisosomes are defined as plasma membrane invaginations filled with protein complexes that are thought to be involved in the desiccation-dehydration processes in eukaryotic organisms with cell walls [87]. These structures have been described for *Trebouxia* cells analyzed within the lichen thallus [87,97,99], and their detection serves as a further confirmation of the desiccation-dehydration tolerance typical of lichens and their phycobionts in particular (e.g., [100,101]).

A further novelty of the present species description is the extensive analysis of the flagellar apparatus of the zoospores. In general, the ultrastructure of the flagellar apparatus is similar to the previously described zoospores of *Asterochloris* *erici* (UTEX 912), *A. glomerata* (UTEX 896 and 894), *A. pyriformis* (UTEX 1712), *Myrmecia israelensis* [102,103], and *Trebouxia impressa* (UTEX 893). All of them showed the peculiar traits described by Melkonian [88] for the motile cells of green algae. Although zoospore formation in cultured *Trebouxia* has been reported several times [88,102,103], the formation of zoospores in culture is not as common as the formation of autospores. Indeed, in culture, autospore formation is the main reproductive strategy adopted by *Trebouxia*. Obtaining zoospores from *T. lynnae* was a very laborious process, and required different treatments (glucose, darkness, etc.), thus the present detailed characterization of their ultrastructure significantly complements the description of the species. Culture growth conditions for inducing the production of zoospores of symbiotic microalgae have not been standardized yet, and future experiments should be centered on this aim.

In this study, we measured and compared the isotopic discrimination of *T. lynnae* and its sister species *T. jamesii*. The results confirm that the pyrenoid is a CCM structure and that *T. lynnae* could be considered to have both C3 and C4 metabolism, as it presents intermediate values (*T. lynnae* −16.96 vs. *T. jamesii* −17.60). This result matches the data obtained by the nuclear genome, in which sequences of proteins involved in carbon uptake mechanisms have been identified [47,48]. Some lichenologists have analyzed isotopic discrimination in whole lichen thalli [62,104,105,106], but these measurements resulted from the photosynthetic characteristics of the photobiont in combination with any potential process happening while lichenized with the mycobiont. Beck et al. [106] measured δ^15^N and δ^13^C patterns in the lichen *Xanthoria parietina* growing on different substrates and in its isolated photobiont (i.e., *Trebouxia decolorans*). The isotope values of *X. parietina* thalli showed a large variability, ranging from −16.0 to 1.2‰ for δ^15^N and from −17.0 to −25.3‰ for δ^13^C. The δ^15^N results for *T. decolorans* showed values ranging between −12.6 and −20.7‰ and the δ^13^C values ranged from −28.1 to −22.5‰. *T. lynnae* showed different values than *T. decolorans*. These preliminary results need further confirmation, as the observed differences may be due to the growth condition of *T. lynnae* used for this experiment. Indeed, the isotopic discrimination analysis requires a lot of biomass, and therefore *T. lynnae* was grown on a medium supplemented with glucose and casein, which induced a faster growth during the standardized period of time (i.e., 21 days) set for sampling the algae for any analyses [18]. Here, the isotopic discrimination analysis was applied for the first time to characterize a lichen-forming microalga to show its potential as the method could be set up using the BBM culture medium and standardized for future studies.

In conclusion, the extensive description provided in this study well supports the new species *Trebouxia lynnae* Barreno as one of the most prominent lichen phycobionts, and stands as a promising model and reference for forthcoming research.

## Figures and Tables

**Table 1:** A comparative table of the morphological and ultrastructural traits of *Trebouxia* species on clade A.

*Trebouxia* Species	Shape and Size of Mature Cells	Chloroplast-Type	Pyrenoid-Type
*Trebouxia aggregata*	Vegetative spherical cells 9–14 μm in diameter [80] Vegetative spherical cells 8–16 (24) μm in diameter [81]	Crenulata-type with slightly branched lobes	Gigantea-type, single pyrenoid
*Trebouxia arboricola*	Vegetative spherical cells 13–15 μm in diameter [80]	Crenulata-type with slightly branched lobes	Gigantea-type, single pyrenoid
*Trebouxia asymmetrica*	Vegetative cells are often ovoid or ellipsoidal, 19 × 14 μm maximum size [82]	Shallowly lobed-type with flat lobe terminations	Gigantea-type, multiple pyrenoids
*Trebouxia crenulata*	Vegetative spherical cells 10–16 (20) μm in diameter [80] Vegetative spherical cells 5–18 (24) μm in diameter [81]	Crenulata-type with branched, tree-like lobes	Crenulata-type, single pyrenoid
*Trebouxia cretacea*	Vegetative spherical cells 15–20 (30) μm in diameter, but also ovoid and ellipsoid cells 20–22 (–30) × 15 μm [83]	Crenulata-type with small unbranched lobes	Gigantea-type, multiple pyrenoids
*Trebouxia decolorans*	Vegetative spherical cells 10–13 (17) μm in diameter [80] Vegetative spherical cells 6–13 (20) μm in diameter [81] Vegetative spherical cells 19–25.5 (30) μm in diameter [84]	Deeply lobed-type	Decolorans-type, multiple pyrenoids
*Trebouxia gigantea*	Vegetative spherical cells 14–22 (27) μm in diameter [80]	Shallowly lobed-type with elongated lobes	Gigantea-type, single pyrenoid
*Trebouxia incrustata*	Vegetative spherical cells 10–14 (15) μm in diameter [80] Vegetative spherical cells 3–10 (22) μm in diameter [81]	Shallowly lobed-type with elongated lobes	Gigantea-type, single pyrenoid
*Trebouxia jamesii*	Vegetative spherical cells 10–15 (20) μm in diameter [80]	Shallowly lobed-type with elongated lobes	Impressa-type, single (or multiple) pyrenoid
***Trebouxia lynnae***	**Vegetative spherical cells 7–12 (16) μm in diameter**	Shallowly lobed-type with elongated lobes	**Impressa-type, single (or multiple) pyrenoid**
*Trebouxia maresiae*	Vegetative spherical cells 7–11 (15) μm in diameter [16]	Crenulata-type with branched tree-like lobes	Maresiae-type, single pyrenoid
*Trebouxia showmanii*	Vegetative spherical cells, often slightly ovoid or ellipsoidal, 11–16 (22) μm in diameter [80]	Crenulata-type with branched, tree-like lobes	Gigantea-type, single pyrenoid
*Trebouxia solaris*	Vegetative spherical cells 15–20 (22) μm in diameter [83]	Crenulata-type	Type not reported, single pyrenoid with starch grains or satellites
*Trebouxia vagua*	Vegetative spherical cells 15–20 μm in diameter, ovoid and ellipsoid cells 20–22 (28) × 13–18 μm [83]	Chloroplast with wide lobes in young cells and narrow lobes in old cells	Type not reported, single pyrenoid with starch grains or satellites

**Table 2:** The characterization of the isotopic composition of *Trebouxia lynnae* sp. nov. compared to *T. jamesii*. The isotopic composition is shown for carbon (δ^13^C) and nitrogen (δ^15^N), and the content of both elements is shown as ‰ of dry mass.

	δ^13^C	δ^15^N	%C	%N
*Trebouxia lynnae*	−16.96‰	6.93‰	47.55	6.41
*Trebouxia jamesii*	−17.60‰	6.43‰	46.95	5.51

## Data Availability

See Table S1.

## Supplementary Materials

The following are available online at https://www.mdpi.com/article/10.3390/biology11081196/s1. Supplementary Table S1: GenBank accession numbers for the species/OTUs included in the phylogenetic analyses. Supplementary Table S2: GenBank accession numbers, host, locality, and reference for the *Trebouxia* species that matched with *Trebouxia* *lynnae* sp. nov. in GenBank.
