# Supplementary material for: Trebouxia lynnae sp. nov. (Former Trebouxia sp. TR9): Biology and Biogeography of an Epitome Lichen Symbiotic Microalga
Source: Biology (Basel). 2022 Aug 10;11(8):1196. doi: 10.3390/biology11081196 (PMC9405249; doi:10.3390/biology11081196)
Supplement: Supplementary file 1 [file biology-11-01196-s001.zip › biology-1802638-supplementary.pdf]

Supplementary Table S1. GenBank accession numbers for the species/OTUs included in the phylogenetic analyses.

Tree Label	OTU / GenBank strain	ITS	cox2	rbcL
A01 (100)	A01 / *Trebouxia* sp. OTU A01 ID 100	KR912355	KR914200	KR914275
A01 (637)	A01 / *Trebouxia* sp. OTU A01 ID 637	KR912367	KR914202	
A02 (8677)	A02 / *Trebouxia* sp. OTU A02 ID 8677	KR912585	KR914206	KR914285
A02 (6018)	A02 / *Trebouxia* sp. OTU A02 ID 6018	KR912646	KR914207	KR914287
A03 (4085)	A03 / *Trebouxia* sp. OTU A03 ID 4085	KR912778	KR914209	KR914290
A03 (6771)	A03 / *Trebouxia* sp. OTU A03 ID 6771	KR912827	KR914210	KR914293
A04 (575)	A04 / *Trebouxia* sp. OTU A04 ID 575	KR913187	KR914231	KR914320
A04 (5041)	A04 / *Trebouxia* sp. OTU A04 ID 5041	KR912904	KR914214	KR914297
A05 (4042)	A05 / *Trebouxia* sp. OTU A05 ID 4042	KR912912	-	KR914299
A05 (8723)	A05 / *Trebouxia* sp. OTU A05 ID 8723	KR912930	-	KR914300
A06 (6621)	A06 / *Trebouxia* sp. OTU A06 ID 6621	KR912987	KR914215	KR914301
A06 (286)	A06 / *Trebouxia* sp. OTU A06 ID 286	KR913027	KR914219	KR914305
A07 (219)	A07 / *Trebouxia* sp. OTU A07 ID 219	KR913043	KR914220	KR914306
A07 (4284)	A07 / *Trebouxia* sp. OTU A07 ID 4284	KR913090	KR914223	KR914310
A08 (5168)	A08 / *Trebouxia* sp. OTU A08 ID 5168	KR913102	KR914225	KR914312
A08 (6024)	A08 / *Trebouxia* sp. OTU A08 ID 6024	KR913123	KR914228	KR914315
A09 (1032)	A09 / *Trebouxia* sp. OTU A09 ID 1032	KR913169	KR914229	KR914317
A09 (906)	A09 / *Trebouxia* sp. OTU A09 ID 906	KR913178	KR914230	KR914318
A10 (575)	A04 / *Trebouxia* sp. OTU A04 ID 575	KR913187	KR914231	KR914320
A11 (527)	A11 / *Trebouxia* sp. OTU A11 ID 527	KR913194	KR914234	KR914323
A11 (493)	A11 / *Trebouxia* sp. OTU A11 ID 493	KR913196	KR914235	KR914324
A12 (736)	A12 / *Trebouxia* sp. OTU A12 ID 736	KR913202	KR914236	KR914326
A12 (127)	A12 / *Trebouxia* sp. OTU A12 ID 127	KR913204	KR914237	KR914327
A13 (L1184)	A13 / *Trebouxia* sp. 1 strain L1184	KJ754304	-	KM091733
A13 (SAG219-1a)	A13 / *Trebouxia arboricola* SAG 219-1a	Z68705	-	AM158960
A13 (AV020)	A13 / *Trebouxia crenulata* voucher AV020	KT819989	-	-
A14 (170)	A14 / *Trebouxia* sp. OTU A14 ID 170	KR913246	KR914256	KR914351
A14 (149)	A14 / *Trebouxia* sp. OTU A14 ID 149	KR913220	KR914242	KR914335
A15 (GIGA)	A15 / *Trebouxia gigantea*	AJ249577	-	-
A15 (UTEX2231)	A15 / *Trebouxia gigantea* UTEX 2231	AF242468	-	-
A15 (197)	A15 / *Trebouxia* sp. OTU A15 ID 197	KR913223	KR914244	KR914337
A16 (901)	A16 / *Trebouxia* sp. OTU A16 ID 901	KR913224	KR914245	KR914338
A16 (452)	A16 / *Trebouxia* sp. OTU A16 ID 452	KR913229	KR914249	KR914342
A17 (6714)	A17 / *Trebouxia* sp. OTU A17 ID 6714	KR913232	KR914250	KR914344
A17 (1602)	A17 / *Trebouxia* sp. OTU A17 ID 1602	KR913235	-	KR914345
A18 (140)	A18 / *Trebouxia* sp. OTU A18 ID 140	KR913237	KR914252	KR914347
A18 (229)	A18 / *Trebouxia* sp. OTU A18 ID 229	KR913240	KR914255	KR914350
A19 (3743)	A19 / *Trebouxia* sp. OTU A19 ID 3743	KR913242	-	-
A20 (147)	A20 / *Trebouxia* sp. OTU A20 ID 147	KR913248	KR914258	KR914352
A21 (334)	A21 / *Trebouxia* sp. OTU A21 ID 334	KR913250	KR914260	KR914354
A21 (335)	A21 / *Trebouxia* sp. OTU A21 ID 335	KR913251	KR914261	KR914355
A22 (007)	A22 / *Trebouxia* sp. OTU A22 ID 007	KR913255	KR914263	KR914359
A22 (031)	A22 / *Trebouxia* sp. OTU A22 ID 031	KR913256	KR914264	KR914360

A23 (SAG2009)	A23 / *Trebouxia showmanii* SAG 2009	FJ626734		
A23 (UTEX2234)	A23 / *Trebouxia showmanii* UTEX 2234	FJ626734	-	AB194857
A25 (6698)	A25 / *Trebouxia* sp. OTU A25 ID 6698	KR913259	KR914265	KR914361
A25 (6703)	A25 / *Trebouxia* sp. OTU A25 ID 6703	KR913260	KR914266	KR914362
A26 (073)	A26 / *Trebouxia* sp. OTU A26 ID 073	KR913261	KR914267	KR914363
A26 (080)	A26 / *Trebouxia* sp. OTU A26 ID 080	KR913262	KR914268	KR914364
A27 (111)	A27 / *Trebouxia* sp. OTU A27 ID 111	KR913263	KR914269	KR914365
A27 (110)	A27 / *Trebouxia* sp. OTU A27 ID 110	KR913264	KR914270	KR914366
A28 (098)	A28 / *Trebouxia* sp. OTU A28 ID 098	KR913265	KR914271	KR914367
A29 (L1739)	A29 / *Trebouxia* sp. clade IV isolate L1739	KJ754246	-	KM091768
A29 (L1141)	A29 / *Trebouxia* sp. clade IV isolate L1141	KJ754245	-	KJ754346
A29 (L1408)	A29 / *Trebouxia* sp. clade IV isolate L1408	KJ754241	-	KJ754347
A30 (036)	A30 / *Trebouxia* sp. OTU A30 ID 036	KR913267	KR914272	KR914369
A31 (030)	A31 / *Trebouxia* sp. OTU A31 ID 030	KR913268	-	-
A32 (075)	A32 / *Trebouxia* sp. OTU A32 ID 075	KR913269	KR914273	KR914370
A33 (P319)	A33 / *Trebouxia decolorans* isolate P-319	AJ970889	-	AM159504
A33 (UTEXB781)	A33 / *Trebouxia decolorans* UTEX B781	FJ626728		
A33 (P121IIcd)	A33 / *Trebouxia* sp. P-121-IIcd	AJ969550	-	AM158967
A34 (6702)	A34 / *Trebouxia* sp. OTU A34 ID 6702	KR913270	-	-
A35 (L1383)	A35 / *Trebouxia arboricola* isolate L1383	KJ754240	-	KM091793
A35 (AV060)		KT819918		
A36 (L101)	A36 / Uncultured *Trebouxia* isolate L-101	AJ969540	-	AJ969664
A37 (L1107)	A37 / *Trebouxia arboricola* isolate L1107	KJ754236	-	-
A38 (Trinkaus356a)	A38 / *Trebouxia* sp. Trinkaus356a	AJ293783	-	-
**A39 (ASUV44; ex *Trebouxia* sp. TR9)**	**A39 / *Trebouxia* sp. TR9 ASUV44**	**KU716051**	**QES94804**	**QHO63910**
A40 (P10)	A40 / *Trebouxia* sp. P-10	AJ969515	-	AJ969659
A41 (9517)	A41 / *Trebouxia vagua* voucher Kirika 9517	MT127650	-	-
A42 (9491)	A42 / *Trebouxia* sp. A42_9491	MT127651	-	-
A43 (9580)	A43 / *Trebouxia* sp. A43_9580	MT127655	-	-
A44 (9714)	A44 / *Trebouxia* sp. A44_9714	MT127659	-	-
A45 (L174)	A45 / Uncultured *Trebouxia* clone L174	KJ576675	-	-
A46 (ASUV142)	A46 / *Trebouxia maresiae* ASUV142	MZ724411	MZ687826	MZ687825
A46 (P280IIaSc)	A46 / *Trebouxia* sp. P-280-IIaSc	AJ969583	-	AJ969660
A47 (G9306)	A47 / Uncultured *Trebouxia* isolate G9306	AJ969505	-	AJ969662
A48 (P133Ia)	A48 / *Trebouxia* sp. P-133-Ia	AJ969551	-	AM158961
A48 (P287VIb)	A48 / *Trebouxia* sp. P-287-VIb	AJ969586	-	AJ969649
A49 (9493)	A49 / *Trebouxia* sp. A49_9493	MT127660	-	-
A50 (L1382)	A50 / *Trebouxia arboricola* isolate L1382	KJ754239	-	-
A51 (P69IaSc)	A51 / *Trebouxia* sp. P-69-IaSc	AJ969534	-	AJ969652
A52 (L2388)	A52 / *Trebouxia* sp. OTU A52 ID L2388	OM275485	-	-
A52 (L2913)	A52 / *Trebouxia* sp. OTU A52 ID L2913	OM275539		-
A53 (16_20)	A53 / *Trebouxia* sp. OTU A53 ID 16 20	OL625070	-	
A53 (16_21)	A53 / *Trebouxia* sp. OTU A53 ID 16 21	OL625071		
A54 (ASUV137)	A54 / *Trebouxia* sp. arnoldoi ASUS137	MT458610		
S10	S10 / *Trebouxia* sp. 1 HAP01	KJ623927	KJ623951	KJ623975

Supplementary Table S2. GenBank accession numbers, host, locality, and reference for the *Trebouxia* species that matched with *Trebouxia lynnae* sp. nov. in GenBank.

GenBank code	Host	Locality/Country
MN684424 Trebouxia sp. TR9 isolate ASV 58	*Ramalina farinacea*	Castellon/Spain
MN684432 Trebouxia sp. TR9 isolate ASV 66	*Ramalina farinacea*	Tenerife-Canary Island/Spain
MN684412 Trebouxia sp. TR9 isolate ASV 46	*Ramalina farinacea*	La Palma-Canary Island/Spain
MN684389 Trebouxia sp. TR9 isolate ASV 23	*Ramalina farinacea*	Tenerife-Canary Island/Spain
MN684454 Trebouxia sp. TR9 isolate ASV 88	*Ramalina farinacea*	Castellon/Spain
MN684473 Trebouxia sp. TR9 isolate ASV 107	*Ramalina farinacea*	Tenerife-Canary Island/Spain
MN684383 Trebouxia sp. TR9 isolate ASV 17	*Ramalina farinacea*	La Palma-Canary Island/Spain
MN684440 Trebouxia sp. TR9 isolate ASV 74	*Ramalina farinacea*	Castellon/Spain
MN684452 Trebouxia sp. TR9 isolate ASV 86	*Ramalina farinacea*	Tenerife-Canary Island/Spain
MN684376 Trebouxia sp. TR9 isolate ASV 10	*Ramalina farinacea*	Castellon/Spain
MN684423 Trebouxia sp. TR9 isolate ASV 57	*Ramalina farinacea*	Tenerife-Canary Island/Spain
MN684429 Trebouxia sp. TR9 isolate ASV 63	*Ramalina farinacea*	La Palma-Canary Island/Spain
MN684438 Trebouxia sp. TR9 isolate ASV 72	*Ramalina farinacea*	Castellon/Spain
MN684419 Trebouxia sp. TR9 isolate ASV 53	*Ramalina farinacea*	Tenerife-Canary Island/Spain
MG687518 Trebouxia sp. BGK-2018	*Lecanographa amylacea*	Białowieża National Park/Poland
MG687515 Trebouxia sp. BGK-2018	*Lecanographa amylacea*	Białowieża National Park/Poland
MG687516Trebouxia sp. BGK-2018	*Lecanographa amylacea*	Białowieża National Park/Poland
MG687517 Trebouxia sp. BGK-2018	*Lecanographa amylacea*	Uppland/Sweden
MG687513 Trebouxia sp. BGK-2018	*Lecanographa amylacea*	Uppland/Sweden
MG687509 Trebouxia sp. BGK-2018	*Lecanographa amylacea*	Uppland/Sweden
MG687508Trebouxia sp. BGK-2018	*Lecanographa amylacea*	Uppland/Sweden
MG687512 Trebouxia sp. BGK-2018	*Lecanographa amylacea*	Uppland/Sweden
MG687507 Trebouxia sp. BGK-2018	*Lecanographa amylacea*	Uppland/Sweden

MG687514 Trebouxia sp. BGK-2018	*Lecanographa amylacea*	Białowieża National Park/Poland
KY066417 Trebouxia sp. 8	*Protoparmelia montagnei*	Almeria/Spain
KY066423 Trebouxia sp. 8	*Protoparmelia montagnei*	Almeria/Spain
KY066421 Trebouxia sp. 8	*Protoparmelia montagnei*	Almeria/Spain
KY066420 Trebouxia sp. 8	*Protoparmelia montagnei*	Almeria/Spain
KY066422 Trebouxia sp. 8	*Protoparmelia montagnei*	Almeria/Spain
KY066424 Trebouxia sp. 8	*Protoparmelia montagnei*	Almeria/Spain
KM369705 Uncultured Trebouxia photobiont clone ARam9	*Ramalina sp.*	New Zealand
KM369706 Uncultured Trebouxia photobiont clone ARam10	*Ramalina sp.*	New Zealand
KF549590 Trebouxia decolorans isolate SCI-03b	*Ramalina menziesii*	California/USA
KF549534 Trebouxia jamesii isolate BA13-01a	*Ramalina menziesii*	California/USA
KF549659 Trebouxia jamesii isolate BA12-03a	*Ramalina menziesii*	California/USA
GU252182.1 Uncultured Trebouxia photobiont clone AL ITS Gu1	*Ramalina farinacea*	Tenerife-Canary Island/Spain
GU252198.1 Uncultured Trebouxia photobiont clone AL ITS Re2	*Ramalina farinacea*	Tenerife-Canary Island/Spain
JN980150 Uncultured Trebouxia photobiont clone S3T	*Ramalina fastigiata*	Spain
JN980147 Uncultured Trebouxia photobiont clone MT	*Ramalina fastigiata*	Spain
JN980148 Uncultured Trebouxia photobiont clone S1	*Ramalina fastigiata*	Spain
EF523354.1 Uncultured Trebouxia photobiont isolate 71	*Ramalina farinacea*	Castellon/Spain
EF523350.1 Uncultured Trebouxia photobiont isolate 67	*Ramalina farinacea*	Castellon/Spain
FJ418565.1 Trebouxia photobiont sp. TR9	*Ramalina farinacea*	Castellon/Spain
KU716051 Trebouxia sp. TR9 26S-18S	*Ramalina farinacea*	Castellon/Spain
